# Association of monetary diet cost of foods and diet quality in Spanish older adults

**DOI:** 10.3389/fpubh.2023.1166787

**Published:** 2023-07-25

**Authors:** Cristina Bouzas, Rosario Pastor, Silvia García, Margalida Monserrat-Mesquida, Miguel Ángel Martínez-González, Jordi Salas-Salvadó, Dolores Corella, Helmut Schröder, J. Alfredo Martínez, Ángel M. Alonso-Gómez, Julia Wärnberg, Jesús Vioque, Dora Romaguera, José Lopez-Miranda, Ramon Estruch, Francisco J. Tinahones, José Lapetra, Lluís Serra-Majem, Blanca Riquelme-Gallego, Anny Romero-Secin, Xavier Pintó, José J. Gaforio, Pilar Matía, Josep Vidal, Miriam Zapatero, Lidia Daimiel, Emilio Ros, Ana García-Arellano, Nancy Babio, Inmaculada Gonzalez-Monje, Olga Castañer, Itziar Abete, Lucas Tojal-Sierra, Juan Carlos Benavente-Marín, Antonio Signes-Pastor, Jadwiga Konieczna, Antonio García-Ríos, Sara Castro-Barquero, José C. Fernández-García, José Manuel Santos-Lozano, Maira Bes-Rastrollo, Cristina Mestres, Patricia Guillem-Saiz, Albert Goday, Leire Goicolea-Güemez, Estanislao Puig-Aguiló, Miguel Ruiz-Canela, Antoni Palau-Galindo, Montse Fitó, Josep A. Tur

**Affiliations:** ^1^CIBER Fisiopatología de la Obesidad y Nutrición (CIBEROBN), Instituto de Salud Carlos III (ISCIII), Madrid, Spain; ^2^Research Group on Community Nutrition and Oxidative Stress, University of Balearic Islands, Palma de Mallorca, Spain; ^3^Health Research Institute of the Balearic Islands (IdISBa), Palma de Mallorca, Spain; ^4^Faculty of Health Sciences, Catholic University of Ávila, Ávila, Spain; ^5^Department of Preventive Medicine and Public Health, IDISNA, University of Navarra, Pamplona, Spain; ^6^Universitat Rovira i Virgili, Departament de Bioquímica i Biotecnologia, Unitat de Nutrició Humana & Institut d'Investigació Sanitària Pere Virgili (IISPV), Reus, Spain; ^7^Department of Preventive Medicine, University of Valencia, Valencia, Spain; ^8^Unit of Cardiovascular Risk and Nutrition, Institut Hospital del Mar de Investigaciones Médicas Municipal d'Investigació Mèdica (IMIM), Barcelona, Spain; ^9^CIBER Epidemiología y Salud Pública (CIBERESP), Instituto de Salud Carlos III (ISCIII), Madrid, Spain; ^10^Cardiometabolics Precision Nutrition Program, IMDEA Food, CEI UAM + CSIC, Madrid, Spain; ^11^Department of Nutrition, Food Sciences, and Physiology, Center for Nutrition Research, University of Navarra, Pamplona, Spain; ^12^Bioaraba Health Research Institute, Osakidetza Basque Health Service, Araba University Hospital, University of the Basque Country UPV/EHU, Vitoria-Gasteiz, Spain; ^13^Department of Nursing, School of Health Sciences, University of Málaga-IBIMA, Málaga, Spain; ^14^Instituto de Investigación Sanitaria y Biomédica de Alicante, Universidad Miguel Hernández, ISABIAL-UMH, Alicante, Spain; ^15^Lipids and Atherosclerosis Unit, Department of Internal Medicine, Maimonides Biomedical Research Institute of Cordoba (IMIBIC), Reina Sofia University Hospital, University of Córdoba, Córdoba, Spain; ^16^Department of Internal Medicine, IDIBAPS, Hospital Clinic, University of Barcelona, Barcelona, Spain; ^17^Department of Endocrinology, Virgen de la Victoria Hospital, University of Málaga, Málaga, Spain; ^18^Department of Family Medicine, Research Unit, Distrito Sanitario Atención Primaria Sevilla, Sevilla, Spain; ^19^Institute for Biomedical Research, University of Las Palmas de Gran Canaria, Las Palmas, Spain; ^20^Department of Preventive Medicine, University of Granada, Granada, Spain; ^21^CIBER Diabetes y Enfermedades Metabólicas (CIBERDEM), Instituto de Salud Carlos III (ISCIII), Madrid, Spain; ^22^Consultorio de Colloto, Centro de Salud Ventanielles, Oviedo, Spain; ^23^Lipids and Vascular Risk Unit, Internal Medicine, Hospital Universitari de Bellvitge, Hospitalet de Llobregat, Barcelona, Spain; ^24^Department of Health Sciences, Center for Advanced Studies in Olive Grove and Olive Oils, University of Jaén, Jaén, Spain; ^25^Department of Endocrinology and Nutrition, Instituto de Investigación Sanitaria San Carlos (IdISSC), Hospital Clínico San Carlos, Universidad Complutense, Madrid, Spain; ^26^Department of Endocrinology, IDIBAPS, Hospital Clinic, University of Barcelona, Barcelona, Spain; ^27^Department of Endocrinology, Hospital La Paz, Madrid, Spain; ^28^Nutritional Control of the Epigenome Group, Precision Nutrition and Obesity Program, IMDEA Food, CEI UAM + CSIC, Madrid, Spain; ^29^Lipid Clinic, Department of Endocrinology and Nutrition, Institut d'Investigacions Biomèdiques August Pi i Sunyer (IDIBAPS), Hospital Clínic, Barcelona, Spain; ^30^Osasunbidea, Servicio Navarro de Salud, Atención Primaria, Pamplona, Spain; ^31^Centro Salud de San Juan, San Juan de Alicante, Spain; ^32^ABS Reus V. Centre d'Assistència Primària Marià Fortuny, Salut Sant Joan de Reus-Baix Camp, Reus, Spain

**Keywords:** monetary cost, Mediterranean diet, provegetarian dietary pattern, dietary inflammatory index, metabolic syndrome

## Abstract

**Background:**

A major barrier to a healthy diet may be the higher price of healthy foods compared to low-quality foods.

**Objectives:**

This study aimed to assess the association between the monetary cost of food and diet quality in Spanish older adults at high risk of cardiovascular disease.

**Methods:**

Cross-sectional analysis was carried out in Spanish older adults (*n* = 6,838; 48.6% female). A validated food frequency questionnaire was used to assess dietary intake. Metabolic syndrome severity, adherence to the Mediterranean diet (MedDiet), adherence to a provegetarian dietary pattern, and dietary inflammatory index were assessed. The economic cost of the foods was obtained from the Spanish Ministry of Agriculture Fisheries and Food database (2015–2017, the period of time when the participants were recruited). The total cost of diet adjusted per 1,000 kcal was computed.

**Results:**

The healthier dietary pattern was associated with a higher cost of the diet. Higher adherence to the MedDiet, anti-inflammatory diet, and the healthy version of the provegetarian dietary pattern were related to higher costs of the diet.

**Conclusion:**

Higher diet quality was associated with a higher dietary cost of the diet per 1,000 kcal/day. Food prices can be an important component of interventions and policies aimed at improving people's diets and preventing diet-related chronic diseases.

**Clinical trial registry number:**

The trial was registered in 2014 at the International Standard Randomized Controlled Trial (ISRCT; http://www.isrctn.com/ISRCTN89898870) with the number 89898870.

## 1. Introduction

The promotion of healthy diets should be part of population strategies to reduce the burden of chronic diseases. Plant-based diets (provegetarian or flexitarian) in which plant-derived foods are preferentially consumed, but not exclusively, have been associated with a lower risk of obesity (1–3), and other diseases, such as type 2 diabetes mellitus (4). An operational definition of a provegetarian diet was initially proposed in the setting of the PREDIMED primary prevention trial, and it was found to be longitudinally associated with lower all-cause mortality (5). The consumption of vegetables, nuts, and whole grains, typical foods of the Mediterranean Diet (MedDiet), was associated with lower levels of inflammation and a lower risk of metabolic syndrome (MetS) (6, 7). An inverse association was also found between adherence to MedDiet and the incidence of the different components of MetS (8, 9). Therefore, the MedDiet has been widely recognized as an optimal eating pattern (balanced, varied, healthy, personalized, appetizing, and functional), and scientific evidence has demonstrated its health benefits, especially in the prevention and control of non-communicable diseases (10–16).

However, a major barrier to eating a healthy diet may be the higher price of these foods. Several studies showed that high adherence to a healthy dietary pattern is associated with higher monetary costs (17–19), and other studies showed that economic constraints lead to the consumption of diets with high energy density (20, 21).

To our knowledge, only one study assessed the association between the cost of diet and the consumption of recommended servings of fruits and vegetables in young adults with MetS (21, 22). However, no studies have been published evaluating the association between diet cost and adherence to MedDiet in older adults with MetS.

This study aimed to assess the association between the monetary cost of food and diet quality in Spanish older non-institutionalized adults with MetS.

## 2. Materials and methods

### 2.1. Study design

A cross-sectional analysis of baseline data of the PREDIMED-Plus trial is shown. The PREDIMED-Plus is an ongoing 6-year, controlled, parallel-group, multicenter, randomized trial conducted in 23 Spanish sites. The trial aimed to assess the effect on cardiovascular disease morbimortality of combined dietary intervention and physical activity. The study protocol can be found at http://predimedplus.com/ (23). The trial was registered at the International Standard Randomized Controlled Trial (ISRCT; http://www.isrctn.com/ISRCTN89898870) with the number 89898870.

### 2.2. Participants, recruitment, and ethics

Eligible participants were community-dwelling adults aged between 55 and 75 years (60–75 for women), with a body mass index (BMI) between 27 and 40 kg/m^2^ and meeting at least three MetS criteria according to the updated harmonized definition of the International Diabetes Federation and the American Heart Association and National Heart, Lung, and Blood Institute (23). Exclusion criteria are available elsewhere (24).

**Figure 1:**
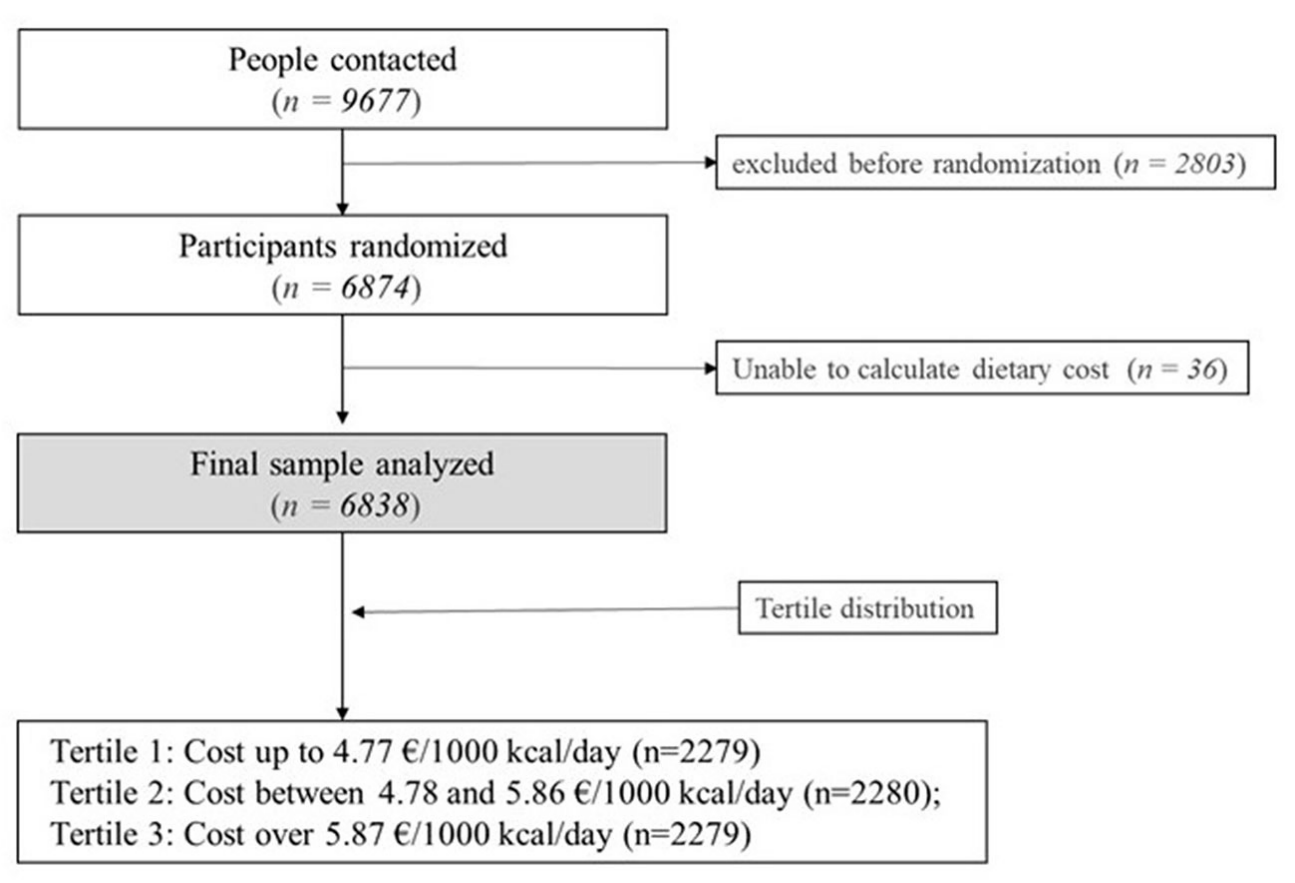
Flow chart of participants.

A total of 9,677 adults were contacted, of which 6,874 met inclusion/exclusion criteria and were included in the study as illustrated by a flow chart (Figure 1). The institutional review boards of the 23 participating centers approved the study protocol, and all participants provided written informed consent.

### 2.3. Dietary assessment

A validated semi-quantitative 143-item food frequency questionnaire (FFQ) was used to assess dietary intake (https://pubmed.ncbi.nlm.nih.gov/20105389/) (25). Trained dietitians administered in person the questionnaire to participants. For each item, the usual portion size was established, and nine consumption frequencies were available, ranging from “never or almost never” to “≥6 times/day”. Food consumption was obtained by adding grams consumed of each item included in the food group. Diet composition was obtained by multiplying the portion size by the frequency and each by the food composition available in Spanish food composition tables (26).

### 2.4. Assessment of the economic cost of diet

The Household Consumption Database of the Spanish Ministry of Agriculture, Fisheries and Food (27) was used to obtain food prices, which were handled similarly to a previous study (28). This dataset includes the average prices of each food per month in the Spanish region. It offers the mean price for the region as a whole, including cities and rural areas. The average price of each food item in Spain between 2015 and 2017 was calculated. When an item of the FFQ included several foods, the average was calculated to estimate the cost of the item as a whole. Then, intakes of each food item (in grams or milliliters) were multiplied by its corresponding price in grams.

Then, the price of the diet was further adjusted by energy density to avoid bias due to amount of food consumed. The more food one eats the more the diet costs. Hence, data were adjusted by energy intake as an indicator of both, the amount of food consumed and energy requirements. Therefore, data in this study are expressed in €/1,000 kcal/day.

It was not possible to calculate the economic cost of the diet for 36 subjects, and accordingly, they were excluded. Therefore, 6,838 subjects were included in this study, as shown in Figure 1. For statistical analysis, participants were classified in tertiles according to their cost of the diet per 1,000 kcal (€/1,000 kcal/day). Tertile 1 included participants expending up to 4.77 €/1,000 kcal/day (*n* = 2,279). Tertile 2 included participants expending between 4.78 and 5.86 €/1,000 kcal/day (*n* = 2,280). Tertile 3 included participants expending over 5.87 €/1,000 kcal/day (*n* = 2,279).

### 2.5. Dietary patterns assessment

Adherence to an energy-reduced MedDiet was assessed by a 17-item Mediterranean diet questionnaire, in which each item is related to a food habit (29). FFQ was used to assess each parameter's intake, and DII was calculated as previously described (30, 31). Negative scores are related to the anti-inflammatory dietary pattern, while positive scores are related to the pro-inflammatory pattern.

Provegetarian dietary patterns were calculated as previously described (32). FFQ was used to assess each parameter's intake. Foods were divided into animal foods, healthy plant foods, and less healthy plant foods. For the assessment of healthy provegetarian food patterns, positive scores were attributed to healthy plant foods, and reverse scores were attributed to the other two groups. For unhealthy provegetarian food patterns, positive scores were attributed to less healthy plant food, and reverse scores to the other two groups. Healthy and unhealthy provegetarian food patterns could both range from 18 to 90.

### 2.6. Health variables

Sociodemographic data (age, sex, educational level, and marital status), as well as smoking habit, medical history, and current medication, were obtained. Blood pressure was measured in triplicate, in a seated position, with a semi-automatic oscillometer (Omron HEM-705CP, Lake Forest, IL, USA). Overnight fasting blood samples were obtained and analyzed by standard enzymatic methods. Total cholesterol, HDL-cholesterol, triglycerides, and fasting plasma glucose were measured. LDL-cholesterol was calculated according to the Friedewald formula. Abdominal obesity was assessed by measuring the waist circumference halfway between the last rib and the iliac crest using an anthropometric tape. The validated Minnesota-REGICOR short physical activity questionnaire assessed physical activity. The metabolic equivalent of task (MET) was calculated by multiplying the minutes spent in each activity by the intensity of the activity. It is shown as energy expenditure in the article (33).

### 2.7. Metabolic syndrome severity score assessment

The metabolic syndrome severity score (MetSSS) was derived from waist circumference, HDL-cholesterol, triglycerides in the blood, blood pressure (systolic and diastolic), and glucose. It was calculated as previously described (34).

### 2.8. Statistics

Statistical analyses were performed with the Statistical Package for Social Sciences version 28.0 (SPSS Inc., Chicago, IL, USA). Data distribution was assessed by Kolmogorov–Smirnov test. Data were expressed as mean and standard deviation (SD) for quantitative variables and sample size and percentage for categorical variables. A *p*-value under 0.01 was considered statistically significant. One-way ANOVA and Bonferroni's *post hoc* analysis were used to assess differences among tertiles of the economic cost of the diet for quantitative variables. Kruskal–Wallis non-parametric tests were also performed for quantitative variables, with Dunn–Bonferroni *post hoc* analysis (data available in Supplementary material). Differences in prevalence among groups were tested using χ^2^. Analysis was furthermore adjusted by sex and education, as they appeared to be confounders for some analysis. Sex was only adjusted by education and vice-versa.

Logistic regression was used to assess the association between several dietary patterns/MetSSS (dependent variable) and the economic cost of the diet (€/1,000 kcal/day) (independent variable). Each dependent variable was categorized into two categories. Cutoff points were percentile 50 for healthy and unhealthy provegetarian dietary patterns and for MetSSS. The cutoff for DII was 0, as it is the cutoff for clinical relevance (30). The cutoff for Mediterranean diet adherence was categorized as low-to-medium adherence (scores 0–10) and high adherence (scores 11–17), as previously described (35). For each variable, crude and adjusted odds ratio (OR) were calculated. We estimated OR (95% confidence intervals) for dietary patterns and MetSSS, both adjusted by sex, age, and educational level.

Pearson's correlations between the cost of the diet and adherence to several dietary patterns were performed. Correlation analysis was adjusted by age, sex, and educational level and is shown as several graphs in the Supplementary material.

## 3. Results

### 3.1. Sociodemographic characteristics of participants according to the economic cost of the diet per 1,000 kcal (€/1,000 kcal/day)

Table 1 shows sociodemographic characteristics and the incidence of MetS components according to the economic cost of the diet per 1,000 kcal. Female participants, as well as those who live alone, were more likely to expend more money on their diet. Lower energy intakes and higher levels of energy expenditure were related to higher costs of the diet per 1,000 kcal/day. Higher education and non-smokers spent more money on their diet. The cost of the diet was directly associated with hyperglycemia and abdominal obesity prevalence and inversely associated with hypertriglyceridemia and low HDL-cholesterol prevalence.

**Table 1:** Sociodemographic characteristics according to the economic cost of the diet per 1,000 kcal.

	**T1^§^(*n* = 2,279)**	**T2^§^(*n* = 2,280)**	**T3^§^(*n* = 2,279)**	***p*-value^‡^^†^**
	**Mean (SD)**	**Mean (SD)**	**Mean (SD)**	
Age (years)	64.7 (5.0)^a^	65.2 (4.9)^a^	65.0 (4.8)	0.006
BMI (kg/m^2^)	32.5 (3.4)	32.5 (3.5)	32.7 (3.5)	0.132
Energy intake (kcal/day)	2,630.3 (684.8)^a, b^	2,436.0 (575.9)^a, c^	2,182.5 (547.2)^b, c^	<0.001
Energy expenditure (kcal/day)	2,323.5 (2,284.3)^b^	2,452.0 (2,286.0)^c^	2,627.5 (2,331.2)^b, c^	<0.001
	*n* (%)	*n* (%)	*n* (%)	
Sex (female)	953 (41.8)	1,097 (48.1)	1,273 (55.9)	<0.001
**Educational level**				
Primary	1,177 (51.6)	1,116 (48.9)	1,057 (46.4)	<0.001
Secondary	676 (29.7)	647 (28.4)	652 (28.6)	
Tertiary	426 (18.7)	517 (22.7)	570 (25.0)	
**Marital status**				
Married	1,753 (77.3)	1,744 (76.6)	1,716 (75.6)	0.113
Divorced/separated	190 (8.4)	170 (7.5)	182 (8.0)	
Widower	211 (9.3)	234 (10.3)	266 (11.7)	
Other (single + religious)	115 (5.1)	129 (5.7)	106 (4.7)	
Living alone^‡^	253 (11.1)	281 (12.3)	323 (14.2)	0.007
**Smoking habit**				
Current smoker	328 (14.5)	276 (12.1)	247 (10.9)	0.005
Former smoker	980 (43.2)	990 (43.6)	992 (43.7)	
Never smoked	959 (42.3)	1,006 (44.3)	1,032 (45.4)	
**MetS components**				
High blood pressure	2,087 (91.6)	2,086 (91.5)	2,104 (92.3)	0.532
Hyperglycaemia	1,667 (73.1)	1,728 (75.8)	1,756 (77.1)	0.008
Hypertriglyceridemia	1,332 (58.4)	1,266 (55.5)	1,205 (52.9)	<0.001
Low HDL-cholesterol	1,044 (45.8)	981 (43.0)	906 (39.8)	<0.001
Abdominal obesity	2,170 (95.2)	2,186 (95.9)	2,216 (97.2)	0.002

BMI, body mass index; HDL-cholesterol, high-density lipoprotein cholesterol; MetS, metabolic syndrome; SD, standard deviation. ^§^Tertiles of the economic cost of the diet per 1,000 kcal: T1: cost up to 4.77 €/day (*n* = 2,279); T2: cost between 4.78 and 5.86 €/day (*n* = 2,280); T3: cost over 5.87 €/day (*n* = 2,279). ^‡^Living alone regardless of marital status. ^**‡**^Differences in means between groups were tested by one-way ANOVA and Bonferroni's *post hoc* (expressed by the letters a, b, and c). Differences in prevalence across groups were examined using χ^2^. ^**†**^Analysis was adjusted by sex and educational level. Sex was only adjusted by educational level and vice-versa.

Adjusted analysis revealed that sex and educational level were confounder factors for most of the sociodemographic variables. Age, energy intake, and expenditure were not related to the economic cost of the diet after adjustment by either or both variables. Moreover, the adjusted analysis revealed that living alone was only relevant for women and those with a middle education level, whereas smoking habit was relevant in the economic cost of the diet for men and with a low-middle education level. Among MetS parameters, hyperglycemia, hypertriglyceridemia, and abdominal obesity prevalence were more related to the economic cost of the diet only for men, while HDL-cholesterol was related to both sexes. Hypertriglyceridemia, HDL-cholesterol, and abdominal obesity were related to the economic cost of the diet only in the lower levels of education (data not shown).

### 3.2. Food intake according to the cost of the diet per 1,000 kcal (€/1,000 kcal/day)

Food intake according to the cost of the diet is shown in Table 2. The higher cost of the diet was related to a higher intake of fruits and vegetables, whole grains, fish and seafood, white and processed meat, coffee and tea, artificially sweetened beverages, and alcoholic drinks. As the cost of the diet was lower, the intake of several foods was higher: potatoes and refined cereals, eggs, milk and dairy, fats, and oils (including olive oil), sweets and pastries, and convenience foods. Cheaper diets were more energetically dense than more expensive diets.

**Table 2:** Food intake according to the economic cost of the diet per 1000 kcal.

	**T1^§^(*n* = 2,279)**	**T2^§^(*n* = 2,280)**	**T3^§^(*n* = 2,279)**	***p*-value^‡^**
	**Mean (SD)**	**Mean (SD)**	**Mean (SD)**	
Energy density (kcal/g)	1.3 (0.2)^a, b^	1.2 (0.2)^a, c^	1.0 (0.2)^b, c^	<0.001
Fruits (g/d)	352.9 (197.1)^a, b^	418.5 (223.0)^a, c^	451.5 (263.3)^b, c^	<0.001
Vegetables (g/d)	271.3 (109.2)^a, b^	329.1 (124.2)^a, c^	393.4 (159.6)^b, c^	<0.001
Potatoes (g/d)	78.7 (50.6)^a, b^	69.8 (43.7)^a, c^	58.0 (40.9)^b, c^	<0.001
Refined cereals (g/d)	152.4 (104.6)^a, b^	109.0 (81.6)^a, c^	74.8 (66.3)^b, c^	<0.001
Whole grains (g/d)	34.7 (70.9)^a, b^	40.9 (62.5)^a^	42.9 (56.6)^b^	<0.001
Legumes (g/d)	20.8 (11.5)	21.0 (11.2)	20.8 (11.7)	0.726
Whitefish (g/d)	26.2 (20.7)^a, b^	38.9 (24.6)^a, c^	48.8 (26.6)^b, c^	<0.001
Bluefish (g/d)	29.2 (19.2)^a, b^	37.8 (22.8)^a, c^	43.4 (25.1)^b, c^	<0.001
Seafood (g/d)	21.1 (16.2)^a, b^	27.8 (19.3)^a, c^	35.5 (27.3)^b, c^	<0.001
White meat (g/d)	54.6 (31.2)^a, b^	62.7 (32.8)^a, c^	68.8 (37.7)^b, c^	<0.001
Red meat (g/d)	50.1 (34.7)	52.4 (35.2)	51.7 (36.1)	0.088
Processed meat (g/d)	33.9 (23.9)^a, b^	36.9 (26.0)^a, c^	39.0 (29.1)^b, c^	<0.001
Eggs (g/d)	25.1 (13.6)^a, b^	24.1 (12.1)^a, c^	23.2 (11.2)^b, c^	<0.001
Milk and dairy (g/d)	361.9 (214.8)^b^	351.0 (204.9)	336.6 (197.9)^b^	<0.001
Nuts (g/d)	14.1 (17.8)^a^	16.6 (18.4)^a^	15.4 (17.4)	<0.001
Olive oil (g/d)	45.0 (17.9)^a, b^	41.3 (16.3)^a, c^	34.1 (15.4)^b, c^	<0.001
vegetable oil (g/d)	2.7 (8.2)^a, b^	1.3 (4.2)^a, c^	0.8 (3.2)^b, c^	<0.001
Other fats (g/d)	3.6 (10.2)^a, b^	2.8 (6.5)^a, c^	1.8 (3.8)^b, c^	<0.001
Sweets and pastries (g/d)	64.4 (53.3)^a, b^	48.8 (38.8)^a, c^	33.9 (30.8)^b, c^	<0.001
Convenience foods (g/d)	25.1 (24.4)^a, b^	22.7 (21.8)^a, c^	20.0 (20.3)^b, c^	<0.001
Coffee and tea (ml/d)	85.0 (58.2)^b^	88.4 (60.9)^c^	94.3 (62.0)^b, c^	<0.001
Sugary beverages (ml/d)	54.0 (112.9)^a, b^	38.5 (84.9)^a^	32.3 (75.8)^b^	<0.001
Artificially sweetened beverages (ml/d)	24.2 (87.6)^b^	27.4 (98.5)^c^	41.5 (129.5)^b, c^	<0.001
Fermented alcoholic beverages (ml/d)	156.2 (231.1)^a, b^	179.7 (257.8)^a^	188.6 (290.8)^b^	<0.001
Distilled spirits (ml/d)	3.1 (9.9)^b^	3.4 (9.9)	4.1 (13.4)^b^	0.010

SD, standard deviation. ^§^Tertiles of the economic cost of the diet per 1,000 kcal: T1: cost up to 4.77 €/day (*n* = 2,279); T2: cost between 4.78 and 5.86 €/day (*n* = 2,280); T3: cost over 5.87 €/day (*n* = 2,279).^‡^Difference in means between groups was tested by one-way ANOVA and Bonferroni's *post hoc* (expressed by the letters a, b, and c).

### 3.3. Association and correlation between the cost of the diet (€/1,000 kcal/day) and adherence to dietary patterns

Tables 3, 4 show the association between the cost of the diet and adherence to dietary patterns. As adherence to MedDiet increases, so does the cost of the diet. Accordingly, more anti-inflammatory patterns are related to the higher cost of the diet. More unhealthy plant-based foods were related to the lower cost of the diet. A higher diet quality, measured either by adherence to MedDiet or DII, was associated with a higher economic cost of the diet per 1,000 kcal/day. High adherence to MedDiet and an anti-inflammatory dietary pattern were more likely when the cost of the diet was higher compared to the lower cost [OR (95% CI): MedDiet: T2: 2.05 (1.75–2.40), T3: 3.59 (3.07–4.18), and anti-inflammatory DII: T2: 1.71 (1.52–1.92), T3: 2.37 (2.10–2.67)].

**Table 3:** Adherence to healthy diets and health status according to the economic cost of the diet per 1,000 kcal.

	**T1^§^(*n* = 2,279)**	**T2^§^(*n* = 2,280)**	**T3^§^(*n* = 2,279)**	***p*-value^‡^**
	**Mean (SD)**	**Mean (SD)**	**Mean (SD)**	
MedDiet (17 items)	7.5 (2.5)^a, b^	8.5 (2.6)^a, c^	9.3 (2.5)^b, c^	<0.001
DII	0.6 (1.9)^a, b^	−0.1 (1.9)^a, c^	−0.5 (2.1)^b, c^	<0.001
Healthy PFP	53.6 (6.4)^a^	54.2 (6.6)^a^	53.8 (6.3)	0.006
Unhealthy PFP	57.8 (6.7)^a, b^	53.5 (6.5)^a, c^	50.6 (6.5)^b, c^	<0.001
MetSSS	3.4 (1.4)^b^	3.5 (1.4)	3.5 (1.4)^b^	0.052

DII, dietary inflammatory index; MedDiet, Mediterranean Diet; MetSSS, metabolic syndrome severity; PFP, provegetarian food pattern; SD, standard deviation. ^§^Tertiles of the economic cost of the diet per 1000 kcal: T1: cost up to 4.77 €/1000kcal/day (*n* = 2,279); T2: cost between 4.78 and 5.86 €/1,000 kcal/day (*n* = 2,280); T3: cost over 5.87 €/1,000 kcal/day (*n* = 2,279).^‡^Differences in means between groups were tested by one-way ANOVA and Bonferroni's *post hoc* (expressed by the letters a, b, and c).

**Table 4:** Association between adherence to healthy diets and health status and the economic cost of the diet per 1,000 kcal.

		**T1^§^(*n* = 2,279)**	**T2^§^(*n* = 2,280)**	**T3^§^(*n* = 2,279)**
		**OR (95% CI)**	**OR (95% CI)**	**OR (95% CI)**
MedDiet	Crude OR	1.00 (ref.)	2.13 (1.81–2.49)^*^	3.80 (3.27–4.43)^*^
	Adjusted OR	1.00 (ref.)	2.05 (1.75–2.40)^*^	3.59 (3.07–4.18)^*^
DII	Crude OR	1.00 (ref.)	1.74 (1.54–1.96)^*^	2.43 (2.16–2.74)^*^
	Adjusted OR	1.00 (ref.)	1.71 (1.52–1.92)^*^	2.37 (2.10–2.67)^*^
Healthy PFP	Crude OR	1.00 (ref.)	1.12 (1.00–1.26)	1.03 (0.91–1.15)
	Adjusted OR	1.00 (ref.)	1.15 (1.02–1.29)^*^	1.10 (0.98–1.24)
Unhealthy PFP	Crude OR	1.00 (ref.)	0.39 (0.35–0.44)^*^	0.18 (0.15–0.20)^*^
	Adjusted OR	1.00 (ref.)	0.32 (0.28–0.37)^*^	0.12 (0.10–0.14)^*^
MetSSS	Crude OR	1.00 (ref.)	0.99 (0.88–1.12)	1.11 (0.98–1.25)
	Adjusted OR	1.00 (ref.)	0.97 (0.86–1.10)	1.05 (0.93–1.19)

CI, confidence interval; MedDiet, Mediterranean Diet; DII, dietary inflammatory index; MetSSS, metabolic syndrome severity; PFP, provegetarian food pattern; OR, odds ratio. ^§^Tertiles of the economic cost of the diet per 1,000 kcal: T1: cost up to 4.77 €/day (*n* = 2,279); T2: cost between 4.78 and 5.86 €/day (*n* = 2,280); T3: cost over 5.87 €/day (*n* = 2,279). Adjusted OR: Adjusted by sex, age, and educational level. ^*^p-value <0.01. Dietary patterns/MetSSS (dependent variable).

Pearson's correlations between the cost of the diet and adherence to several dietary patterns were performed as graphs (see Supplementary material). The cost of the diet positively correlated with MedDiet adherence and inversely correlated with unhealthy provegetarian dietary patterns and with DII. In other words, adherence to MedDiet increased when the cost of the diet increased. As the cost of the diet increased, the unhealthy provegetarian dietary pattern decreased. The inflammatory potential of the diet became more anti-inflammatory when the cost of the diet increased.

## 4. Discussion

Current findings showed that higher diet quality, measured either by adherence to MedDiet, by adherence to a healthy provegetarian dietary pattern, or by a low inflammatory index, was associated with a higher diet cost per 1,000 kcal/day. These results showed in previous studies on the adult population are consistent with those of the current study with older adults, which shows the strength of these findings (36–39).

In addition to other factors that may influence diet quality, such as nutrition knowledge, food accessibility and availability, current results confirm that economic resources, and food prices can be an important component of interventions and policies aimed at improving population diets and preventing diet-related chronic diseases (40, 41). Healthier diets at lower costs could be achieved by purchasing foods in a street market (42).

The scientific literature reflected estimates of savings in medical care that would mean greater adherence to the MedDiet. Thus, in an economic model published in 2019, those results showed that increasing the percentage of the population adhering to MedDiet by 20% produced annual savings in cardiovascular disease-related costs in the United States and Canada. Moreover, when adherence increased to 80%, savings continued to increase proportionately (43).

A positive association between the cost of the adjusted diet per 1,000 kcal and the prevalence of abdominal obesity was also observed. However, the current analysis adjusted for sex and educational level showed that this association was only relevant for men. When the analysis delved into the reasons for this positive association between the prevalence of abdominal obesity and the cost of diet in men, it was found that current participants who were abdominally obese at the beginning of the study consumed higher amounts of processed meats and alcoholic beverages, both fermented and distilled, than those who did not show abdominal obesity (data not shown). Our results show that participants with higher monetary expenditure on foods also had the lowest energy density in their diet. The group with the higher expenditure consumed more alcoholic beverages; however, they also had more fruits, vegetables, and meat, which are products more expensive than others. Furthermore, the “cheaper” diets were more energy dense and included more foods with a high energy density and low nutritional value such as sugary sweetened beverages, sweets, pastries, and convenience foods. When the association between diet cost and some food groups was evaluated, processed meats and alcoholic beverages were associated with higher monetary expenditures. Moreover, it cannot rule out the existence of reverse causation because of the cross-sectional design. A previous study associated protein intake with a higher cost of the diet, regardless of overall diet quality. Nevertheless, the source of the protein consumed seemed to have an impact on diet quality. Animal protein sources with lower salt, saturated fat, and added sugars were associated with higher diet quality (42).

The literature showed that the usual consumption of light to moderate alcoholic beverages, mainly red wine, is associated with a lower risk of total mortality, coronary artery disease, diabetes mellitus, congestive heart failure, and stroke. However, higher levels of alcohol consumption were associated with increased cardiovascular risk (43). Several studies showed that light to moderate alcohol consumption was not associated with increased adiposity, while excessive alcohol consumption was more related to weight gain (44). Previous studies showed a positive association between the consumption of processed meats and the incidence of obesity and central obesity (45).

In a study conducted with Spanish adults in 2016 (46), no positive association was found between the cost of the energy-adjusted diet, weight changes, and BMI, which coincided with those found in this study. The authors then reported an association of increased diet cost with a decrease in body weight and BMI, but these associations were no longer present when the models were adjusted for energy density. Current results are also in agreement with previous findings from a Spanish cohort where participants who spent more money on food adjusted by energy intake had higher BMI (46). On the other hand, a previous study performed in Japan did not find any association between caloric expenditure and the energy-adjusted cost of the diet (47). This was not the case for our participants. The higher the money spent on food (adjusted by energy density), the higher the calories spent on physical activity. Even though this variable was estimated from a questionnaire, a clinically relevant difference was found in that analysis. Socioeconomic status plays a role in health (48, 49), which might explain why people who spend more money on food have more money available because they belong to a higher socioeconomic status. Higher socioeconomic status is also related to higher levels of physical activity (50).

In this study, the cost of the diet was inversely proportional to the prevalence of hypertriglyceridemia and low HDL-cholesterol per 1,000 kcal. The adjusted analysis showed that hypertriglyceridemia was related to the economic cost of diet in men and low levels of education, while HDL-cholesterol was related to the economic cost of diet in both sexes and low educational levels. These results agree with those previously published that showed that lower cardiovascular risk was associated with greater adherence to healthy eating patterns (1–3, 8, 9). The current and previous studies both show that healthy dietary patterns are more expensive.

This study showed that women living alone and subjects with higher education had higher costs of their diet adjusted for 1,000 kcal. The adjusted analysis also revealed that sex and educational level were confounding factors for most of the outcomes. Other previous studies conducted on adults showed the same positive association between diet cost and educational level (51, 52). Published studies also reported sex differences when adjusting the cost of the diet per 1,000 kcal (52–54). However, when the current cost of the diet according to sex without adjusting for energy was assessed, it was observed that men had more dietary expenditure than women (data not shown). This pattern likely reflects that energy intake and diet are related. In this sense, the current results showed that the lower energy intakes were related to a higher cost of the diet per 1,000 kcal, but after the adjustment considering the covariates of sex and education, this relationship ceased to be significant.

Previous studies conducted in children or youth populations associated a healthier diet with a greater cost. The low sociocultural and socioeconomic level of the parents was related to a lower amount of money spent on food, and therefore, to unhealthier diets (48, 49). Healthier and more expensive diets in children were achieved by a varied diet, richer in fish and fruits, with a lower energy density (48). However, this association was true for poor or moderate diet quality, when measured by adherence to MedDiet. Within the range of high diet quality, the cost was similar (49). Therefore, MedDiet can be a dietary pattern easy to maintain and cost-limited when adherence is high.

Finally, the current higher cost of the diet was related to higher consumption of some food characteristics of MedDiet, such as fruits and vegetables, whole grains, and fish and seafood. These results are in line with those shown in previous studies (22, 55). It is important to note that not all foods with high nutrient density have a high cost; so, it is possible to design a diet following the Mediterranean dietary pattern using the lowest monetary cost options in each food group. It should be highlighted the importance of legumes as a good nutrient quality and a low-price food group in the context of a healthy dietary pattern. Moreover, only the healthy provegetarian dietary pattern was not associated with dietary costs, in agreement with a previous study conducted in Spain (56). It was the only index that was not significant, as those participants with the highest adherence to the MedDiet spent on average 1.42 €/day more than those with the poorest adherence.

### 4.1. Strengths and limitations

This study provides information on the monetary costs of the diet and their association with the quality of the diet measured by different indices. Currently, this issue is gaining relevance in public health worldwide, due to the current economic crisis in many Mediterranean countries, with the need for designing nutritional education programs that integrate healthy diets with more profitable options.

The main limitation of this study is that the level of economic income of the participants was not analyzed, so it was not possible to analyze its relationship with the quality of the diet. Such an analysis would have been interesting, as several studies have previously shown, that economic constraints lead to the consumption of less healthy diets that are characterized by high energy density (20, 21). Another limitation was the validity and reliability of dietary consumption data from surveys, being well known for the potential for bias (57–59). Moreover, FFQ does not include all specifications of each product consumed, which only allows us to calculate a median of cost between similar products that match the FFQ category. For example, under the category of sweetened beverages, there are several brands usually consumed and several flavors, which differ in cost. Average prices for Spain between 2015 and 2017 were used because the participants in the study were recruited in this period. A final limitation is that this study was observational and cross-sectional, and its design does not allow for establishing causal inferences; hence, only associations were made.

## 5. Conclusion

Higher diet quality, as measured by adherence to the MedDiet or a low inflammatory index, was associated with a higher dietary cost per 1,000 kcal/day. Lower dietary expenditures were associated with a higher prevalence of hypertriglyceridemia and low HDL-cholesterol levels. Therefore, food prices can be an important component of interventions and policies aimed at improving people's diets and preventing diet-related chronic diseases.

## Data availability statement

The raw data supporting the conclusions of this article will be made available by the authors, without undue reservation.

## Ethics statement

The studies involving human participants were reviewed and approved by Research Ethics Committee from all recruitment centers approved the study protocol, according to the ethical standards of the Declaration of Helsinki. All participants provided written informed consent. All centers have the ethics approval and consent from all the Ethics Committee, and within them, the Ethics Committee of Research of Balearic Islands (ref. CEIC-IB2251/14PI). The patients/participants provided their written informed consent to participate in this study.

## Author contributions

CB and RP conducted the statistical analyses and drafted the manuscript. CB, RP, and JT made substantial contributions to the conception and design of the manuscript. All authors contributed substantially to the acquisition of data or analysis and interpretation of data, revised the manuscript critically for important intellectual content, and approved the final version to be published.

## Supplementary material

Supplementary Figure 1 Correlation between dietary cost and MedDiet adherence.

Supplementary Figure 2 Correlation between dietary cost and MedDiet adherence.

Supplementary Figure 3 Correlation between dietary cost and MedDiet adherence.

Supplementary Table 1 Sociodemographic characteristics according to economic cost the diet per 1,000 kcal.

Supplementary Table 2 Food intake according to economic cost of diet per 1,000 kcal.

Supplementary Table 3 Adherence to healthy diets and health status according to economic cost of the diet per 1,000 kcal.
