# Supplementary figures and images for: Association of monetary diet cost of foods and diet quality in Spanish older adults
Source: Front Public Health. 2023 Jul 25;11:1166787. doi: 10.3389/fpubh.2023.1166787 (PMC10408666; doi:10.3389/fpubh.2023.1166787)

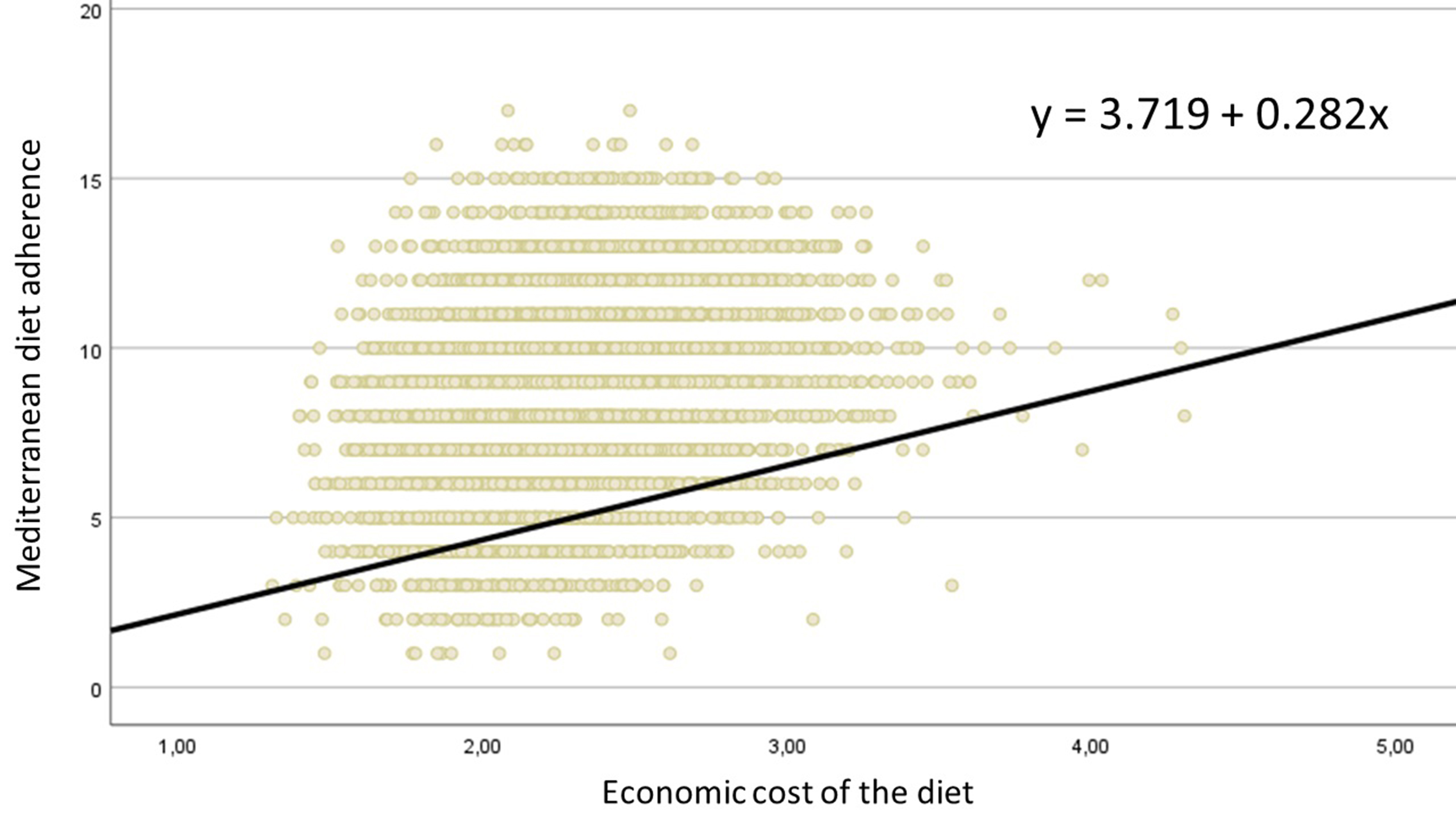

Supplement: Supplementary Figure 1 — Correlation between dietary cost and MedDiet adherence. [file Image_1.jpg]

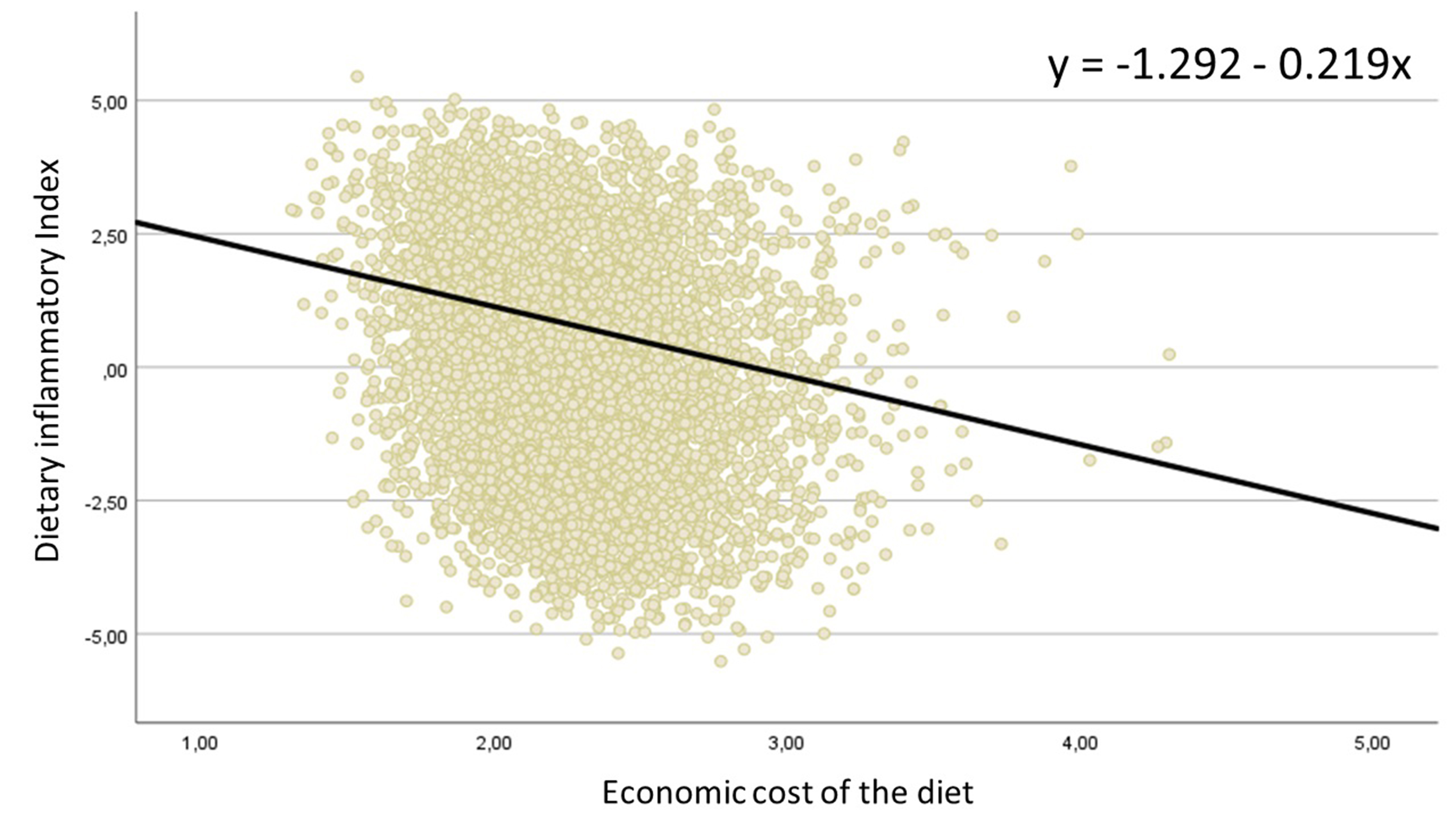

Supplement: Supplementary Figure 2 — Correlation between dietary cost and MedDiet adherence. [file Image_2.jpg]

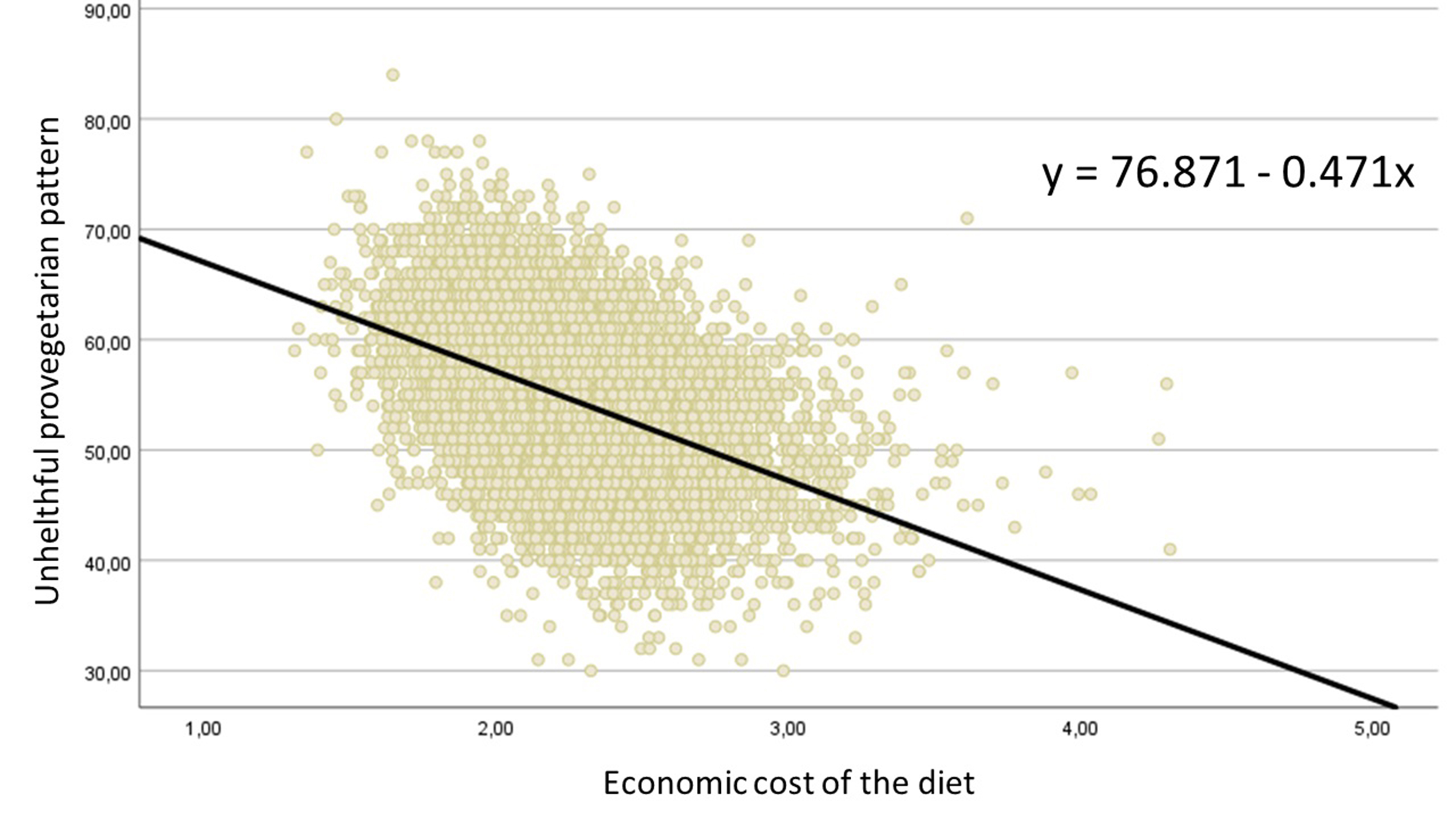

Supplement: Supplementary Figure 3 — Correlation between dietary cost and MedDiet adherence. [file Image_3.jpg]
