# Supplementary material for: Association of monetary diet cost of foods and diet quality in Spanish older adults
Source: Front Public Health. 2023 Jul 25;11:1166787. doi: 10.3389/fpubh.2023.1166787 (PMC10408666; doi:10.3389/fpubh.2023.1166787)
Supplement: Supplementary Table 1 — Sociodemographic characteristics according to economic cost the diet per 1,000 kcal. [file Table_1.docx]

**Supplementary Table 1**. Sociodemographic characteristics according to economic cost the diet per 1000 kcal.

|  | T1 §  (*n* =2279) | T2 §  (*n* =2280) | T3 §  (*n* =2279) | p-value **‡ †** |
| --- | --- | --- | --- | --- |
|  | Median (IQR) | Median (IQR) | Median (IQR) |  |
| Age (years) | 65.0 (8.0) ^a^ | 65.0 (8.0) ^a^ | 65.0 (7.0) | 0.008 |
| BMI (kg/m^2^) | 32.1 (5.1) | 32.0 (5.2) | 32.4 (5.0) | 0.078 |
| Energy intake (kcal/day) | 2554.1 (826.9) ^a,b^ | 2384.6 (712.5) ^a,c^ | 2136.4 (701.0) ^b,c^ | <0.001 |
| Energy expenditure (kcal/day) | 1706.3 (2377.6) ^b^ | 1830.4 (2540.3) ^c^ | 2042.0 (2699.3) ^b,c^ | <0.001 |
|  | n (%) | n (%) | n (%) |  |
| Sex (female) | 953 (41.8) | 1097 (48.1) | 1273 (55.9) | <0.001 |
| Educational level |  |  |  |  |
| Primary | 1177 (51.6) | 1116 (48.9) | 1057 (46.4) | <0.001 |
| Secondary | 676 (29.7) | 647 (28.4) | 652 (28.6) |  |
| Tertiary | 426 (18.7) | 517 (22.7) | 570 (25.0) |  |
| Marital status |  |  |  |  |
| Married | 1753 (77.3) | 1744 (76.6) | 1716 (75.6) | 0.113 |
| Divorced/separated | 190 (8.4) | 170 (7.5) | 182 (8.0) |  |
| Widower | 211 (9.3) | 234 (10.3) | 266 (11.7) |  |
| Other (single + religious) | 115 (5.1) | 129 (5.7) | 106 (4.7) |  |
| Living alone ‡ | 253 (11.1) | 281 (12.3) | 323 (14.2) | 0.007 |
| Smoking habit |  |  |  |  |
| Current smoker | 328 (14.5) | 276 (12.1) | 247 (10.9) | 0.005 |
| Former smoker | 980 (43.2) | 990 (43.6) | 992 (43.7) |  |
| Never smoked | 959 (42.3) | 1006 (44.3) | 1032 (45.4) |  |
| MetS components |  |  |  |  |
| High blood pressure | 2087 (91.6) | 2086 (91.5) | 2104 (92.3) | 0.532 |
| Hyperglycaemia | 1667 (73.1) | 1728 (75.8) | 1756 (77.1) | 0.008 |
| Hypertriglyceridemia | 1332 (58.4) | 1266 (55.5) | 1205 (52.9) | <0.001 |
| Low HDL-cholesterol | 1044 (45.8) | 981 (43.0) | 906 (39.8) | <0.001 |
| Abdominal obesity | 2170 (95.2) | 2186 (95.9) | 2216 (97.2) | 0.002 |

**Abbreviations**: BMI: Body Mass Index. HDL-cholesterol: High density lipoprotein cholesterol. MetS: Metabolic Syndrome. SD. Standard deviation. ^§^Tertiles of economic cost of the diet per 1000 kcal: T1: Cost up to 4.77 €/day (n=2279); T2: Cost between 4.78 and 5.86 €/day (n=2280); T3: Cost over 5.87 €/day (n=2279). ^‡^Living alone regardless of marital status. **^‡^**Differences in means between groups were tested by Kruskal-Wallis and Dunn-Bonferroni’s post-hoc (expressed by the letters a, b, c). Differences in prevalence’s across groups were examined using χ^2^. **^†^**Analysis was adjusted by sex and educational level. Sex was only adjusted by educational level, and vice-verse.
