# Supplementary material for: Association of monetary diet cost of foods and diet quality in Spanish older adults
Source: Front Public Health. 2023 Jul 25;11:1166787. doi: 10.3389/fpubh.2023.1166787 (PMC10408666; doi:10.3389/fpubh.2023.1166787)
Supplement: Supplementary Table 2 — Food intake according to economic cost of diet per 1,000 kcal. [file Table_2.docx]

**Supplementary Table 2**. Food intake according to economic cost of diet per 1000 kcal.

|  | T1 §  (*n* =2279) | T2 §  (*n* =2280) | T3 §  (*n* =2279) | p-value **‡ †** |
| --- | --- | --- | --- | --- |
|  | Median (IQR) | Median (IQR) | Median (IQR) |  |
| Energy density (kcal/g) | 1.3 (0.3) ^a,b^ | 1.2 (0.2) ^a,c^ | 1.0 (0.2) ^b,c^ | <0.001 |
| Fruits (g/d) | 324.6 (254.3) ^a,b^ | 385.5 (282.3) ^a,c^ | 410.4 (297.0) ^b,c^ | <0.001 |
| Vegetables (g/d) | 255.5 (138.6) ^a,b^ | 314.5 (161.6) ^a,c^ | 368.9 (207.2) ^b,c^ | <0.001 |
| Potatoes (g/d) | 85.7 (64.3) ^a,b^ | 61.3 (64.3) ^a,c^ | 41.9 (60.5) ^b,c^ | <0.001 |
| Refined cereals (g/d) | 187.5 (128.6) ^a,b^ | 92.1 (155.4) ^a,c^ | 66.4 (75.0) ^b,c^ | <0.001 |
| Whole grains (g/d) | 0.0 (32.1) ^a,b^ | 0.0 (75.0) ^a^ | 13.0 (75.0) ^b^ | <0.001 |
| Legumes (g/d) | 17.1 (13.1) | 20.6 (13.1) | 17.1 (13.1) | 0.676 |
| White fish (g/d) | 21.4 (11.4) ^a,b^ | 21.4 (42.9) ^a,c^ | 64.3 (42.9) ^b,c^ | <0.001 |
| Blue fish (g/d) | 25.7 (21.4) ^a,b^ | 30.1 (37.6) ^a,c^ | 40.0 (38.5) ^b,c^ | <0.001 |
| Seafood (g/d) | 17.3 (17.3) ^a,b^ | 30.6 (17.3) ^a,c^ | 30.6 (28.6) ^b,c^ | <0.001 |
| White meat (g/d) | 64.3 (52.8) ^a,b^ | 64.3 (42.9) ^a,c^ | 64.3 (42.9) ^b,c^ | <0.001 |
| Red meat (g/d) | 42.8 (44.3) | 44.7 (49.5) | 42.8 (51.0) | 0.058 |
| Processed meat (g/d) | 29.4 (25.2) ^a,b^ | 32.4 (26.5) ^a,c^ | 32.4 (24.8) ^b,c^ | <0.001 |
| Eggs (g/d) | 25.7 (0.0) ^a,b^ | 25.7 (0.0) ^a,c^ | 25.7 (0.0) ^b,c^ | <0.001 |
| Milk and dairy (g/d) | 310.2 (306.6) ^b^ | 309.3 (282.5) | 305.1 (195.0) ^b^ | 0.009 |
| Nuts (g/d) | 8.0 (19.1) ^a^ | 10.6 (21.7) ^a^ | 8.6 (23.6) | <0.001 |
| Olive oil (g/d) | 50.0 (25.0) ^a,b^ | 50.0 (25.0) ^a,c^ | 25.0 (25.0) ^b,c^ | <0.001 |
| Vegetal oils (g/d) | 0.0 (1.4) ^a,b^ | 0.0 (0.7) ^a,c^ | 0.0 (0.7) ^b,c^ | <0.001 |
| Other fats (g/d) | 0.0 (5.1) ^a,b^ | 0.0 (2.5) ^a,c^ | 0.0 (1.6) ^b,c^ | <0.001 |
| Sweets and pastries (g/d) | 53.3 (57.4) ^a,b^ | 40.1 (45.3) ^a,c^ | 26.9 (35.0) ^b,c^ | <0.001 |
| Convenience foods (g/d) | 20.7 (27.2) ^a,b^ | 18.6 (25.1) ^a,c^ | 15.4 (21.0) ^b,c^ | <0.001 |
| Coffee and tea (ml/d) | 64.0 (75.0) ^b^ | 71.4 (75.0) ^c^ | 100.0 (75.0) ^b,c^ | <0.001 |
| Sugary beverages (ml/d) | 13.3 (48.5) ^a,b^ | 0.0 (28.6) ^a^ | 0.0 (28.6) ^b^ | <0.001 |
| Artificially sweetened beverages (ml/d) | 0.0 (0.0)^,b^ | 0.0 (13.3) ^c^ | 0.0 (13.3) ^b,c^ | <0.001 |
| Fermented alcoholic beverages (ml/d) | 61.4 (193.3) ^a,b^ | 82.3 (235.4) ^a^ | 74.7 (233.8) ^b^ | <0.001 |
| Distilled spirits (ml/d) | 0.0 (3.3) | 0.0 (3.3) | 0.0 (3.3) | 0.136 |

**Abbreviations**: IQR: Interquartile range. ^§^Tertiles of economic cost of the diet per 1000 kcal: T1: Cost up to 4.77 €/day (n=2279); T2: Cost between 4.78 and 5.86 €/day (n=2280); T3: Cost over 5.87 €/day (n=2279). **^‡^**Difference in means between groups were tested Kruskal-Wallis and Dunn-Bonferroni’s post-hoc (expressed by the letters a, b, c).
