# Supplementary material for: Association of monetary diet cost of foods and diet quality in Spanish older adults
Source: Front Public Health. 2023 Jul 25;11:1166787. doi: 10.3389/fpubh.2023.1166787 (PMC10408666; doi:10.3389/fpubh.2023.1166787)
Supplement: Supplementary Table 3 — Adherence to healthy diets and health status according to economic cost of the diet per 1,000 kcal. [file Table_3.docx]

**Supplementary Table 3.** Adherence to healthy diets and health status according to economic cost of the diet per 1000 kcal.

|  | T1 §  (*n* =2279) | T2 §  (*n* =2280) | T3 §  (*n* =2279) | p-value **‡ †** |
| --- | --- | --- | --- | --- |
|  | Median (IQR) | Median (IQR) | Median (IQR) |  |
| MedDiet (17 item) | 7.0 (3.0) ^a,b^ | 8.0 (3.0) ^a,c^ | 9.3 (2.5) ^b,c^ | <0.001 |
| DII | 0.7 (2.8) ^a,b^ | -0.1 (3.0) ^a,c^ | -0.5 (2.1) ^b,c^ | <0.001 |
| Healthful PFP | 53.0 (9.0) ^a^ | 54.0 (8.0) ^a^ | 53.8 (6.3) ^b,c^ | 0.031 |
| Unhealthful PFP | 58.0 (9.0) ^a,b^ | 54.0 (9.0) ^a,c^ | 50.6 (6.5) ^b,c^ | <0.001 |
| MetSSS | 3.3 (1.9) ^b^ | 3.3 (1.9) | 3.5 (1.4) ^b^ | 0.044 |

**Abbreviations**: DII: Dietary inflammatory index. MedDiet: Mediterranean Diet. MetSSS: Metabolic syndrome severity. PFP: Provegetarian food pattern. IQR: Interquartile range. ^§^Tertiles of economic cost of the diet per 1000 kcal: T1: Cost up to 4.77 €/1000kcal/day (n=2279); T2: Cost between 4.78 and 5.86 €/1000 kcal/day (n=2280); T3: Cost over 5.87 €/1000kcal/day (n=2279). **^‡^**Differences in means between groups were tested by Kruskal-Wallis and Dunn-Bonferroni’s post-hoc (expressed by the letters a, b, c).
